# Molecular characterization of Indian pathotypes of *Puccinia striiformis* f. sp. *tritici* and multigene phylogenetic analysis to establish inter- and intraspecific relationships

**DOI:** 10.1590/1678-4685-GMB-2017-0171

**Published:** 2018-09-21

**Authors:** Rashmi Aggarwal, Deepika Kulshreshtha, Sapna Sharma, Vaibhav K. Singh, Channappa Manjunatha, Subhash C. Bhardwaj, Mahender S. Saharan

**Affiliations:** ^1^ Fungal Molecular Biology Laboratory, Division of Plant Pathology, Indian Agricultural Research Institute, New Delhi, India; ^2^ ICAR- Indian Agricultural Research Institute, Regional Station Wellington, Tamilnadu, India; ^3^ Indian Institute of Wheat and Barley Research, Regional Station, Flowerdale, Shimla, Himachal Pradesh, India

**Keywords:** Wheat, stripe rust, virulence, diversity, phylogeny

## Abstract

Stripe rust caused by *Puccinia striiformis* f. sp. *tritici* (Pst) is one of the most devastating diseases of wheat (*Triticum* spp.) worldwide. Indian isolates were characterised based on their phenotypic reaction on differential hosts carrying different *Yr* genes. Based on virulence/avirulence structure, isolates were characterised into ten different pathotypes *viz.* 70S0-2, 67S64, 70S4, 66S0, 70S64, 66S64-1, 38S102, 47S102, 46S119, and 78S84. These Indian pathotypes of *P. striiformis* f. sp. *tritici* and 38 pathotypes of other rust species (*P. graminis tritici* and *P. triticina*) were used in this study to analyze their molecular phylogenetic relationship. The nucleotides of rDNA-ITS, partial β*-tubulin* and *ketopantoate reductase* genes of all the pathotypes were sequenced directly after PCR. Based on sequence data of rDNA-ITS and β*-tubulin*, three phylogenetic groups corresponding to three different species of *Puccinia* were obtained. Asian isolates formed a distinct evolutionary lineage than from those derived from USA. The sequence similarity of Indian pathotypes with other Asian (China and Iran) isolates indicated the same origin of pathotypes. The results will allow rapid identification of Indian *P.striiformis* f. sp. *tritici* pathotypes causing stripe rust in wheat, assist in making predictions regarding potential rust pathotypes, and identifying sources of resistance to the disease in advance.

## Introduction

Wheat is one of the most important cereal crops in the world and serves as staple food for billions of people. It is the second major cereal crop after rice both in area of production and consumption, and plays a vital role in food and nutritional security. Rusts are among the most widespread and economically important diseases of cereal crops (Roelfs *et al.*, 1992). These obligate parasites are highly specialized and unveil significant variation in the pathogen population (Singh *et al.*, 2008). Mehta (1941) reported economic losses due to rusts up to Rs. 60 million annually and Prasada (1965) estimated losses of Rs. 392 million in India. The requirement of wheat production in the year 2050 is projected at 120 million tons considering its growing demand for consumption and trade due to bursting population (Singh *et al.*, 2014). Stripe rust is the most damaging and important disease challenging wheat production worldwide including India (Wellings, 2011). The most serious constraint to protect yield and productivity enhancement that has emerged in the last few years is stripe rust (yellow rust) susceptibility of commercially available wheat cultivars.

Stripe rust in wheat is caused by *Puccinia striiformis* f.sp. *tritici* (Pst) and it is present in most wheat-growing regions of the world. In some cases, if infection occurs at early stage and weather remains favorable up to adult stage, stripe rust can cause up to 100% loss (Syed *et al.*, 2007). It is thought that long-distance dispersal by wind might play a key role in the dissemination of the disease. Widespread occurrence of stripe rust was observed in sub-mountainous districts of Punjab on the widely cultivated wheat variety PBW343 during 2008-09 and the disease was as high as 60 – 80% resulting in drastic reduction in yield and replacement of this cultivar. During 2010–2011, stripe rust appeared in severe form in plain areas in Jammu &Kashmir, foot hills of Punjab and Himachal Pradesh, parts of Haryana, and tarai regions of Uttarakhand (Sharma and Saharan, 2011).

Recently, stripe rust has gained importance in India particularly in North Western Plain Zone (NWPZ) and Northern Hills Zone (NHZ) (Prashar *et al.*, 2007; Saharan *et al.*, 2013). During 2008, the disease occurred in moderate to severe form in Punjab and caused losses of approximately Rs 236 x10^7^(Jindal *et al.*, 2012). *P. striiformis* f. sp. *tritici* was commonly assumed to have a macrocyclic lifecycle but with missing pycnial and aecial stages until very recently when it was shown to be able to infect some *Berberis* species (Jin *et al.*, 2010). Under natural conditions, the role of sexual reproduction in the evolution of the pathogen in the US Pacific Northwest and India is limited (Wang *et al.*, 2015), as *Berberis* role is not ascertained. More than 140 races of *Puccinia striiformis* f. sp*. tritici* have been identified in the US and a total of 28 pathotypes have been reported in India (Line and Qayoum, 1992; Chen *et al.*, 2010; Tomar *et al.*, 2014; Bhardwaj *et al.*, 2014). Genetic resistance, i.e. growing resistant cultivars through deployment of stripe rust resistance genes (*Yr* gene), is the most economical, effective and environmentally friendly approach to control the disease (Wellings, 2011). The existence of a large number of pathotypes/races shows the rapid evolution of the pathogen virulence and selections by host crop cultivars with various resistance genes. This continued evolution of rust pathogens against known resistant sources resulted in several yellow rust resistance genes in wheat since 1966; until now, more than 70 catalogued genes have been identified (Talha *et al.*, 2016; Chen and Kang 2017). The predominance of Pst races with virulence for *Yr2* in the 1970s, *Yr9* in the 1990s, and *Yr27* in recent years contributed to large regional epidemics and crop losses (Chen, 2005; Wellings, 2010).

The genetic diversity of *Pst* has been investigated since 1990’s using various molecular techniques. For *Pst* population in US, a high genetic diversity was found among 115 single-spore isolates using the random amplified polymorphic DNA (RAPD) technique (Chen *et al.*, 1993). Later, isolates collected from the south central US since 2000 were found to be genetically distinct from older isolates (collected before 2000) using the amplified fragment length polymorphic (AFLP) technique (Markell and Milus, 2008). More recently, phenotypic and genotypic diversity studies using simple sequence repeat (SSR) markers in relatively small or large stripe rust epidemic regions in Northwest China suggested extensive genetic recombination in the Chinese population (Mboup *et al.*, 2009; Lu *et al.*, 2011). In contrast, relatively low genetic diversities have been reported using AFLP markers for the Australian and European *Pst* populations as the pathogen appears to be clonal (Hovmøller *et al.*, 2002; Enjalbert *et al.*, 2005; Hovmøller and Justesen 2007). No study has been reported on genetic diversity in Indian races of *P. striiformis tritici*, therefore this study was conducted to analyze evolution and genetic variations of *P. striiformis tritici* pathotypes prevalent in India. Furthermore, genetic diversity at an interspecific level was also studied using multigene sequence analysis.

In this study, nucleotide sequence data for rDNA-ITS regions, partial β-tubulin, and *ketopantoate reductase* genes of 10 Pst pathotypes along with isolates of other *Puccinia* species were analyzed. The first objective was to characterize the Indian pathotypes of *Puccinia striiformis tritici* and assess the relationship among them. The second objective was to clarify the phylogenetic relationships among three *Puccinia* species infecting wheat and to investigate whether DNA sequence data support the taxonomic separation of these three species.

## Material and Methods

### Collection and maintenance of *P. striiformis tritici* (Pst)

A large number of wheat samples infected with *P. striiformis tritici* were collected from different wheat growing zones of the country. Single urediniospore cultures were maintained on susceptible genotype ‘Agra local’ under glass house conditions. Urediniospores were collected on sterilized butter paper and stored at -40°C for further use.

### Virulence analysis of Pst isolates

The single uredinial isolates of different rust samples mentioned above were tested for avirulence/virulence on seedlings of differential hosts having different resistance genes. The differential hosts were grown in 10-cm diameter pots and inoculated at single leaf seedling stage, keeping three replicates per differential per isolate. The inoculation and disease recording was done as per standard procedure (Nagarajan *et al.*, 1983; Bhardwaj *et al.*, 2012).

### DNA extraction and template preparation

DNA was extracted from 100 mg of urediniospores of each pathotype using ZR soil microbe DNA miniprep kit (Zymo research, Irvine, CA, USA) following manufacturer’s protocol. The DNA obtained was stored at -20°C. One microliter of ribonuclease at10 mg/mL was added to the extracted nucleic acid and kept at 4°C overnight to completely digest the RNA. DNA was quantified by the Nanodrop spectrophotometer (ND1000, Waltham, MA, USA) and DNA concentration was finally adjusted to 50 ng/μLfor PCR amplification.

### Primer design and PCR amplification

To determine the phylogenetic relationship among 10 different pathotypes, the primers from the two genes (β *tubulin* and *ketopantoate reductase*) along with ITS1-5.8S-ITS2 region (Table S1; White *et al.*, 1990) were designed using software Primer3. Primer quality was checked using IDT-oligoanalyser software to check the potential of secondary structures, self primer dimer and hetero primer dimer formation within and between the different primer sets. The PCR assays were performed in 25 L volume containing 75 ng of genomic DNA of each isolate, 200 μm each dNTP, 0.2 μM primer, 1.5 mM MgCl_2_, 2.5 U *Taq* DNA polymerase, and 1 X *Taq* buffer in thermal cycler (Bio-Rad, Hercules, CA, USA) programmed for one cycle of denaturation at 94 °C for 4 min followed by 35 cycles of denaturation at 94 °C for 1 min, annealing at 55-62°C for 1 min, extension at 72 °C for 2 min, and final extension at 72 °C for 7 min.

The amplified products were resolved by electrophoresis in 1.2% agarose gels run at 80 V for 2 h in TAE buffer (1X) and stained with ethidium bromide at 0.5 μg/mL. The gels were visualized under UV light and photographed with gel documentation unit (Syngene Inc, Cambridge, UK). PCR fragments were excised from agarose gels and purified using QIAquick gel extraction kit (Qiagen). Amplified products were sequenced by ABI 3100 Genetic analyzer at the Department of Biochemistry, South Campus, Delhi University, India. Sequences were BLAST analysed using NCBI database to check for their specificity.

### Data analysis

For phylogenetic analysis, sequences were manually aligned using Bioedit ver. 7.0.9 (Hall, 1999) to remove ambiguous base and primer sequences. Multiple alignments were performed in ClustalW. The sequences obtained for *ketopentaote reductase* gene, β *tubulin* gene and the ITS region were trimmed, aligned, and the phylogenetic tree was prepared using the software Mega 6 (Tamura *et al.*, 2013). For a comparison with results from earlier research, rDNA-ITS and β *tubulin* sequence data of *P. striiformis tritici*, *P. graminis tritici*, and *P. triticina* from NCBI GenBank were used in a phylogenetic analysis (Table S2).Two *Uromyces* sequences of ITS regions and the β *tubulin* from GenBank were used as outgroups in ITS and β *tubulin* phylogenetic analysis. As no sequences were available for the *ketopentaote reductase* gene of Pgt and Pt, we characterized the Indian pathotypes of Pst only. The reference sequence of Pst was taken from the *ketopentaote reductase* gene sequences to analyse the phylogenetic evolution of the *P*. *striiformis* f. sp. *tritici* pathotypes in India.

The evolutionary history was inferred using the NJ method. All positions containing gaps and missing data were eliminated from the dataset (complete deletion option). The evolutionary distances were computed using the maximum composite likelihood method. The Jaccard similarity matrices were used to perform cluster analyses using the neighbor joining (NJ) procedure. Support for the clusters was evaluated using boot strapping analyses with 1000 iterations. Nonparametric bootstrap (BS) was used to assess support for branching topologies (Felsenstein, 1978). The final trimmed sequences were submitted in NCBI database for accession numbers.

## Results

### Virulence behavior

Virulence analysis of single urediniospore cultures of various samples resolved into 10 pathotypes based on avirulence/virulence behavior on differential hosts (Table 1). Among these 10 pathotypes, predominant pathotypes 78S84 and 46S119 were virulent to *Yr2, Yr6, Yr7, Yr8, Yr9, Yr17, Yr18, Yr19, Yr21, Yr22, Yr23, Yr25, Yrso* and avirulent to *Yr1, Yr5, Yr10, Yr11, Yr12, Yr13, Yr14, Yr15, Yr16, Yr24, Yr26, Yrsk* genes. Pathotype 38S102 isolated in 1973 from Nilgiri Hills (SHZ) showed avirulence to *Yr9* along with*Yr*1, *Yr*3, *Yr*5,*Yr*10, *Yr*11, *Yr*12, *Yr*13, *Yr*14, *Yr*15, *Yr*16, *Yr*24, *Yr*26, *Yr*sp, *Yr*sk genes and virulence to *Yr*2, *Yr*4, *Yr*6, *Yr*7, *Yr*8, *Yr*17, *Yr*18, *Yr*19, *Yr*21, *Yr*22, *Yr*23, *Yr*25, *Yr*A genes. Pathotypes 70S0-2, 70S4, 67S64, and 66S0 detected during 1936-37 nearly showed a similar behavior in knocking down R-genes (Table 1).

**Table 1:** Avirulence/virulence formulae for Indian *Puccinia striiformis* f. sp. *tritici* pathotypes used in the study

S. No.	Name of pathotype (Old name)	Name of pathotype (New name)	Place	Year of Detection	Virulent/Avirulent on wheat variety	Avirulent for *Yr* gene	Virulent for *Yr* gene
1	19	70S0-2	Rawalpindi (Pakistan)	1936	Kalyansona/Sonalika	*Yr*2, *Yr*3, *Yr*4, *Yr*8, *Yr*9, *Yr*10, *Yr*11, *Yr*12, *Yr*13, *Yr*14, *Yr*15, *Yr*16, *Yr*24, *Yr*25, *Yr*26 *Yr*sp *Yr*sk	*Yr*1, *Yr*7, *Yr*18, *Yr*19, *Yr*21, *Yr*22, *Yr*23
2	31	67S64	Shimla, HP	1936	Kalyansona/Sonalika	*Yr*3, *Yr*4, *Yr*9, *Yr*10, *Yr*11, *Yr*12, *Yr*13, *Yr*14, *Yr*15, *Yr*16, *Yr*24, *Yr*25, *Yr*26 *Yr*sp *Yr*sk	*Yr*1, *Yr*2, *Yr*7, *Yr*8, *Yr*18, *Yr*19, *Yr*21, *Yr*22, *Yr*23
3	A	70S4	Gurdaspur	1937	Kalyansona/Sonalika	*Yr*2, *Yr*3, *Yr*4, *Yr*9, *Yr*10, *Yr*11, *Yr*12, *Yr*13, *Yr*14, *Yr*15, *Yr*16, *Yr*24, *Yr*25, *Yr*26 *Yr*sp *Yr*sk	*Yr*1, *Yr*7, *Yr*8, *Yr*18, *Yr*19, *Yr*21, *Yr*22, *Yr*23
4	14	66S0	Kangra	1937	Kalyansona/Sonalika	*Yr*1, *Yr*2, *Yr*3, *Yr*4, *Yr*8, *Yr*9, *Yr*10, *Yr*11, *Yr*12, *Yr*13, *Yr*14, *Yr*15, *Yr*16, *Yr*24, *Yr*25, *Yr*26 *Yr*sp *Yr*sk	*Yr*7, *Yr*18, *Yr*19, *Yr*21, *Yr*22, *Yr*23 *Yr*A
5	20A	70S64	Punjab	1970	Kalyansona/Sonalika	*Yr*1 *Yr*3, *Yr*4, *Yr*9, *Yr*10, *Yr*11, *Yr*12, *Yr*13, *Yr*14, *Yr*15, *Yr*16, *Yr*24, *Yr*25, *Yr*26 *Yr*sp *Yr*sk	*Yr*2, *Yr*7, *Yr*8, *Yr*18, *Yr*19, *Yr*21, *Yr*22, *Yr*23 *Yr*A
6	38A	66S64-1	Punjab	1970	Kalyansona/Sonalika	*Yr*1 *Yr*3, *Yr*4, *Yr*8, *Yr*9, *Yr*10, *Yr*11, *Yr*12, *Yr*13, *Yr*14, *Yr*15, *Yr*16, *Yr*24, *Yr*25, *Yr*26 *Yr*sp *Yr*sk	*Yr*2, *Yr*7, *Yr*18, *Yr*19, *Yr*21, *Yr*22, *Yr*23 *Yr*A
7	I	38S102	Nilgiri hills	1973	Avir. *Yr9*	*Yr*1, *Yr*3, *Yr*5, *Yr*9 *Yr*10, *Yr*11, *Yr*12, *Yr*13, *Yr*14, *Yr*15, *Yr*16, *Yr*24, *Yr*26 *Yr*sp *Yr*sk	*Yr*2, *Yr*4, *Yr*6, *Yr*7, *Yr*8, *Yr*17, *Yr*18, *Yr*19, *Yr*21, *Yr*22, *Yr*23, *Yr*25 *Yr*A
8	K	47S102	Punjab	1982	Kalyansona/Sonalika (*Yr2*)	*Yr4, Yr5, Yr9 Yr10, Yr11, Yr12, Yr13, Yr14, Yr15, Yr16, Yr24, Yr26, Yrsp, Yrso, Yrsk*	*Yr1, Yr2, Yr3Yr6, Yr7, Yr8, Yr17, Yr18, Yr19, Yr21, Yr22, Yr23, Yr25, YrA, Yrsd, Yrso*
9	*Yr9* pt.	46S119	Gurdaspur	1996	CPAN 3004, HS240	*Yr1, Yr5, Yr10, Yr11, Yr12, Yr13, Yr14, Yr15, Yr16, Yr24, Yr26, Yrsp, Yrso , Yrsk*	*Yr2, Yr3, Yr4, Yr6, Yr7, Yr8, Yr9, Yr17, Yr18, Yr19, Yr21, Yr22, Yr23, Yr25, YrA, Yrsd, Yrso*
10	PBW343 pt.	78S84	Batala (Gurdaspur) Punjab	2001	PBW343 (*Yr9*, *Yr27*)	*Yr1, Yr4, Yr5, Yr10, Yr11, Yr12, Yr13, Yr14, Yr15, Yr 16, Yr24, Yr26, Yrsk, YrA, Yrsd*	*Yr2, Yr6, Yr7, Yr8, Yr9, Yr17, Yr18, Yr19, Yr21, Yr22, Yr23, Yr25, Yr27 Yrso*

### ITS sequence-based analysis

**Figure 1:**
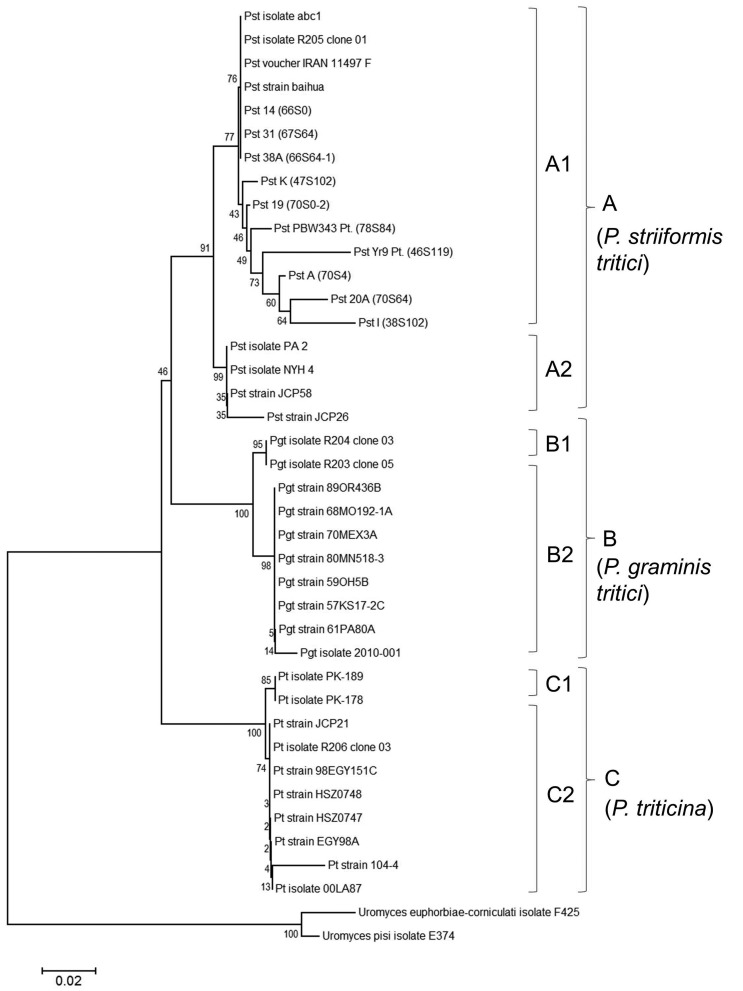
Neighbor-joining phylogenetic tree based on nucleotide sequence of ITS showing phylogenetic relations among pathotypes of *P. striiformis tritici*, *P. graminis tritici*, and *P. triticina*.

A sharp band of about 600 bp was amplified in all the pathotypes taken for the study using ITS specific primers (Figure S1a). In the phylogenetic tree (Figure 1), three main groups could be distinguished: group A, included all pathotypes of *P. striiformis*; group B was closely related to group A, and included all *P. graminis tritici* isolates in this group, while group C was distinct from groups A and B, comprising all *P. triticina* isolates.

Furthermore, Group A was divided into two subgroups (A1 and A2). In subgroup A1 the isolates were from Asian countries (Iran, China, and India), whereas all the isolates of USA were clustered into subgroup A2. In subgroup A1, the two primitive Indian pathotypes 67S64 (1936) and 66S0 (1937) and another pathotype 66S64-1 (1970) were clustered with Iran and Chinese isolates showing the diverse nature of these pathotypes. Pathotype 38S102 and 70S64 were found to be closely related in phylogeny and both these pathotypes originated at the same time in India *i.e.,*~1970.The multiple sequence alignment of all the Indian pathotypes separately for ITS1 (Figure S2) and ITS2 (Figure S3) regions showed that ITS2 region was more conserved than the ITS1. Significant divergence was observed among positions 11–155 within the ITS1 sequences.

The base sequence at 52,123,146, and 150 of ITS1 region separated the old pathotypes (70S0-2, 70S4, 67S64, and 66S0) originated in 1936-37 from the recent ones (46S119 and 78S84), whereas nucleotides at 123 and 146 positions showed variation in presently prevalent pathotypes 46S119 and 78S84.The 5.8S rRNA gene was found to be fully conserved among all the 10 pathotypes. The ITS2 region was also mostly conserved; however, at position 17 and 18, pathotype 38S102 showed nucleotide substitution from AT to TA. Insertion and deletion events were also observed in this region.

Group B included all isolates of *P. graminis tritici* collected from Iran and the US and two subgroups (B1 and B2) could be distinguished. Subgroup B1 included two *P. graminis tritici* sequences from Iran, while US isolates were included in subgroup B2. In group C, two subgroups (C1 and C2) of *P. triticina* were formed; two Pakistani isolates PK-178 and PK-189 also belonged to the same subgroup (C1). Subgroup C2 included all the US isolates except R206 (Belgium) and 104-4 (India).

### *Ketopantoate reductase* gene sequence-based analysis

**Figure 2:**
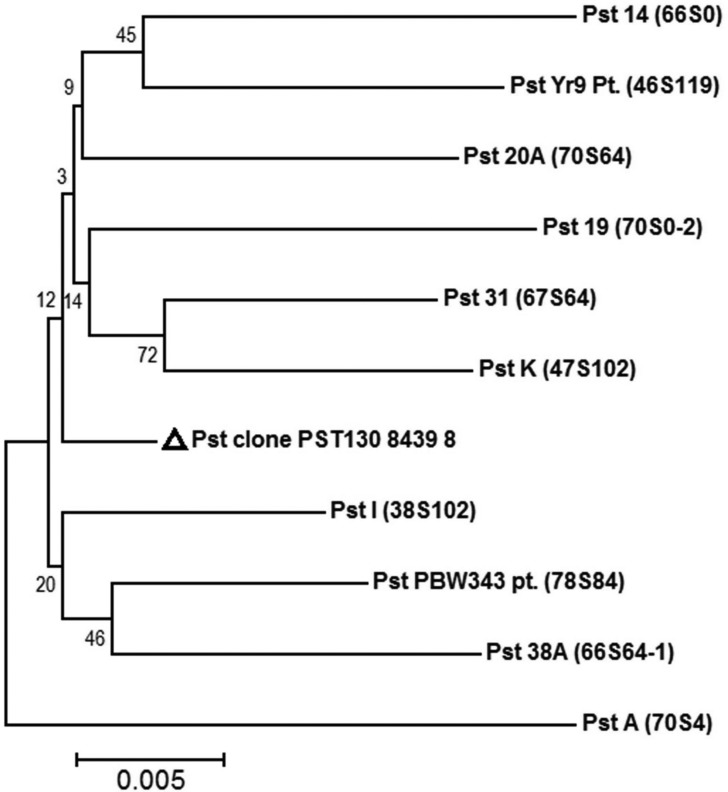
Neighbor-joining phylogenetic tree based on nucleotide sequence of *ketopantoate reductase* gene showing phylogenetic relations among *P. striiformis* f. sp. *tritici* pathotypes.

A band of 1500 bp was obtained using *ketopantoate reductase* specific primers (Figure S1b). The phylogenetic analysis of sequences of *ketopantoate reductase* gene was performed with pathotypes collected from different regions of India. In the phylogeny, pathotype 70S4 detected in year 1937 from Gurdaspur, singly formed a separate cluster (Figure 2), whereas all the remaining pathotypes (70S0-2, 67S64, 66S0, 70S64, 66S64-1, 38S102, 47S102, 46S119, and 78S84) grouped together into a separate major cluster. The major cluster was further subdivided into two sub clusters, wherein the only pathotype PST-130 from Washington, USA used as reference for the *ketopantoate reductase* phylogeny grouped together with Indian pathotypes. The sequences were very closely related and showed 0.0005 base substitutions/site.

### β-*tubulin* gene sequence-based analysis

**Figure 3:**
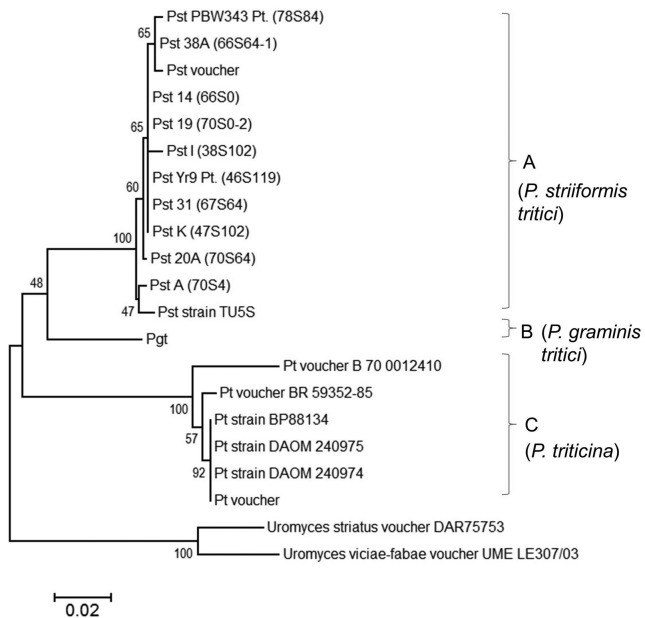
Neighbor-joining tree based on nucleotide sequence of β*-tubulin* showing phylogenetic relations among pathotypes of *P. striiformis tritici*, *P. graminis tritici*, and *P.triticina*

For β-tubulin specific primers, the desired band of 957 bp was obtained in all the pathotypes of *P. striiformis tritici* (Figure S1c). Based on β*-tubulin* gene sequence analysis of all the pathotypes, two major clusters were formed (Figure 3) showing close similarity between all Pst pathotypes, which were grouped together in same sub cluster while Pt strains were in a separate sub cluster. Overall, the sequences of β*-tubulin* gene have changed during the course of evolution and the phylogeny distinguished the three rust fungi *viz*. Pst, Pgt, and Pt into three separate groups *viz*, group A, B, and C, respectively. Group A clustered Pst pathotypes and showed that Pathotype 70S4 had sequence homology with the Chinese pathotype (Pst TU5S).The Indian pathotype 78S84 detected in 2001, an old Indian pathotype (66S64-1) detected in the year 1970, and the voucher pathotype of Sydney also clustered together in the same group. Similarly, pathotypes 67S64, 47S102, and 46S119 were found closely related in the phylogeny. The interspecies analysis of Pst and Pt strains separated the isolates of the two species into separate clusters.

### Multiple gene-based phylogeny using combined rDNA-ITS, partial β*-tubulin*, and *ketopantoate reductase* gene sequences data

**Figure 4:**
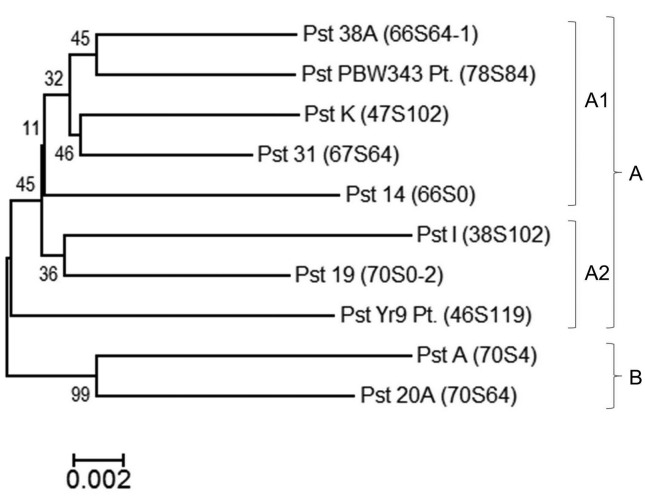
Neighbor-joining tree based on combined nucleotide sequence of ITS, β*-tubulin*, and *ketopantoate reductase* showing phylogenetic relations among pathotypes of *P. striiformis tritici*.

The accession numbers of ITS, partial β*-tubulin*, and *ketopantoate reductase* gene submitted to NCBI database for each pathotype are given in Table 2. A phylogenetic tree was constructed from the combined rDNA-ITS, partial β*-tubulin*, and *ketopantoate reductase* gene sequence data sets of Indian pathotypes (Figure 4). The pathotypes clustered in a manner that showed the evolution of new pathotypes from the previously existing ones. Pathotype 38S102 was clustered with pathotype 70S0-2 and pathotype 70S4 with pathotype 70S64.Two clusters were formed, of which cluster A was the major cluster further subdivided into A1 and A2, consisting mostly of *P. striiformis tritici* pathotypes taken for the study.

**Table 2:** Indian Pst pathotype sequences of ITS and β-*tubulin* and *ketopantoate reductase* submitted to NCBI for phylogenetic study.

Past Pathotypes	ITS	β-*tubulin*	*ketopantoate reductase*
19 (70S0-2)	KX061103	KX424983	KX249826
31 (67S64)	KT320894	KX424984	KX249827
A (70S4)	KT320892	KX424987	KX249833
14 (66S0)	KX061104	KX424982	KX249825
20A (70S64)	KT320893	KX424988	KX061102
38A (66S64-1)	KT305926	KT345695	KX249832
I (38S102)	KT320895	KX424981	KX249830
K (47S102)	KT320891	KT345694	KX249829
*Yr9* pt. (46S119)	JQ360861	KX424986	KX249828
PBW343 pt. (78S84)	JQ360860	KX424985	KX249831

## Discussion

A revolution in the analysis of plant pathogens diversity came with PCR-based molecular techniques, which has enhanced the understanding of taxonomy and population structure. Sequence based molecular characterization may help in understanding the genetic structure and relationship among the races of plant pathogens. It has been reported earlier that genetic variation in populations of rust pathogens are shaped by sexual recombination (Kolmer, 1992), mutation (Park *et al.*, 2002), migration of genetically distinct individuals between and within crop production regions, genetic drift extinction events (Justesen *et al.*, 2002). Furthermore, agricultural practices, such as the cultivation of varieties having different R-genes, promoted the selection of virulent types (Burdon and Silk, 1997).

Virulence phenotypes have been correlated with the genetic differentiation of *Puccinia triticina* populations based on SSR variations (Kolmer and Ordoñez, 2007). Cryptic sexual reproduction or gene conversion can also have a drastic effect on the reshuffling of genetic material (Burt *et al.*, 1996; Bengtsson, 2003) resulting in faster spread of virulent genes and emergence of new virulence combinations. In India, sexual reproduction in wheat rust pathogens is not reported so far, therefore new pathotypes originate either through mutation or parasexuality. Early molecular studies provided evidence of genetic distinction of three *Puccinia* species on wheat, but the phylogenetic relationships among pathotypes belonging to these three species have not been reported and were addressed in this study.

Historically, the use of major race specific resistance (*R*) genes in wheat varieties has been an effective method for disease management. However, these approaches are hampered by the evolution of resistance-breaking races of Pst. In recent years, concerns over stripe rust have increased with the emergence of new and more aggressive Pst races that have expanded the virulence profiles and are capable of adapting to warmer temperatures compared to most previous races (Hovmøller *et al.*, 2010). For example, the appearance of Pst races that overcome widely deployed *R* genes (such as *Yr2*, *Yr9*, *Yr17,* and *Yr27*) has led to destructive pandemics (Wellings, 2011). In the present study, inter and intraspecific phylogenetic relationship among Indian pathotypes of *Puccinia* species infecting wheat has been established based on multigene sequence analysis. The understanding of pathogenicity, avirulence/virulence behavior, and their evolution is critical for the development of more effective breeding strategies and achieve durable resistance.

Molecular analyses were initiated to study the phylogenetic connection and to provide evidence for reassessment of the *Puccinia* species generic taxonomy. We selected multiple genes to detect genetic variation among *P. striiformis* pathotypes from different regions because this method is technically simple and fast. ITS and β*-tubulin* sequences have been widely used for phylogenetic studies; however, recently, a diagnostic marker has been developed for Pst using *ketopantoate reductase* gene (Aggarwal *et al.*, 2017). Therefore, we used this gene to analyse the evolutionary relationship among Pst pathotypes.

Based on the ITS sequence analysis, the pathotype 38S102 formed a separate cluster. Position 17 and 18 of ITS2 region might explain the different behavior of this pathotype and separated it from other Pst pathotypes. Similarly, the nucleotide substitution at position 123 and 146 of pathotype 78S84 might explain the virulence of this pathotype for *Yr27* (Prashar *et al.*, 2007). Furthermore, the sequence similarity of Indian pathotypes with other Asian (China and Iran) isolates suggests the intrinsic ability of Pst for long distance spore dispersal (Brown and Hovmøller, 2002) and these new races pose an increasing threat to global wheat production and food security (Milus *et al.*, 2009). Our results demonstrated that multigene analysis is useful for detection of genetic variation among *P. striiformis* isolates.

There was considerable sequence variation in the ITS sequences and little or no variation in the regions of the 5.8S rDNA. The sequence variations of ITS reported in the literature are usually higher in ITS1 than ITS2 (Park *et al.*, 2001). Our data showed that the ITS rRNA gene can be used for inferring the phylogeny of closely related species, as well as for examining the relationships between and within the populations of the same species. This may also be helpful for the identification of species that cannot be distinguished using only morphological characteristics, or for testing interbreeding potential. In order to improve the use of the ITS region for diagnostics in fungi, the secondary structure of ITS2 can be used to increase the specificity of ITS spacers (Landis and Gargas, 2007).Whole genome based evolutionary analysis has also been performed previously, which separated Pst pathotypes on the basis of their year of detection and geographical region (Kiran *et al.*, 2017).

In our results, the ITS phylogenetic tree best represented all the three fungi in accordance with their detection time and geographical location. However, the phylogenetic tree based on multigene analysis explained the clustering of Pst pathotypes on a broader aspect of their evolution from previously existing pathotypes due to the selection pressure. In the present study, ITS and β*-tubulin* sequences separated the three rust fungi of wheat into separate clusters; therefore, these can be used as markers to identify and differentiate this fungus. Earlier, a RAPD and URP-based molecular analysis has been done for predominant Indian pathotypes of all the three rust species infecting wheat (Aggarwal *et al.*, 2018). However, this is the first report of a multigene sequence-based phylogenetic analysis of Pst pathotypes along with *P. graminis tritici* and *P. triticina*.

This study will allow rapid identification of Indian *Puccinia striiformis* f. sp. *tritici* pathotypes causing stripe rust in wheat and the resulting phylogeny will assist in making predictions regarding potential rust pathotypes of wheat and identifying sources of disease resistance in advance. Our results indicate the need for more detailed phylogenetic analyses within the Pst clades, which will require the inclusion of additional taxa and loci.

## Supplementary Material

The following online material is available for this article:

Figure S1 - Agarose gel showing amplified products.

Figure S2 - Multiple sequence alignment of internal transcribed spacer 1.

Figure S3 - Multiple sequence alignment of internal transcribed spacer 2.

Table S1 - Details of genes and primers.

Table S2 - NCBI sequences of ITS, β-tubulin, and ketopantoate reductase
