# Supplementary material for: Molecular characterization of Indian pathotypes of Puccinia striiformis f. sp. tritici and multigene phylogenetic analysis to establish inter- and intraspecific relationships
Source: Genet Mol Biol. 2018 Sep 21;41(4):834–42. doi: 10.1590/1678-4685-GMB-2017-0171 (PMC6415613; doi:10.1590/1678-4685-GMB-2017-0171)
Supplement: Supplementary file 1 [file 1415-4757-GMB-1678-4685-GMB-2017-0171-s004.pdf]

Supplementary Material to "Molecular characterization of Indian pathotypes of *Puccinia striiformis* f. sp. *tritici* and multigene phylogenetic analysis to establish inter- and intraspecific relationships"

Table S1 - Details of genes and primers taken for amplification of Indian *Puccinia striiformis* f. sp. *tritici* pathotypes.

Gene	Forward primer (5'->3')	Reverse primer (5'->3‘)	Product length (bp)	Tm (°C)
β-tubulin	GGGTGCTGGTAACAACTGGG	CCGTGAATTGATCGGCCAC	957	60.1
ketopantoate reductase	ATCACGAAATCCTCCAAAAATC	CTACTTTATGTCAGTGTCT	1500	62.8
**ITS**	TCCGTAGGTGAACCTGCGG	TCCTCCGCTTATTGATATGC	600	55.0
