# Supplementary material for: Molecular characterization of Indian pathotypes of Puccinia striiformis f. sp. tritici and multigene phylogenetic analysis to establish inter- and intraspecific relationships
Source: Genet Mol Biol. 2018 Sep 21;41(4):834–42. doi: 10.1590/1678-4685-GMB-2017-0171 (PMC6415613; doi:10.1590/1678-4685-GMB-2017-0171)
Supplement: Supplementary file 2 [file 1415-4757-GMB-1678-4685-GMB-2017-0171-s005.pdf]

Supplementary Material to "Molecular characterization of Indian pathotypes of *Puccinia striiformis* f. sp. *tritici* and multigene phylogenetic analysis to establish inter- and intraspecific relationships"

Table S2 - NCBI sequences of ITS, β-tubulin, and ketopantoate reductase used for phylogenetic study

**Species**	**Isolate**	**Location**	**GenBank accession no.**		
			ITS	*β-tubulin*	*Ketopantoate reductase*
P. striiformis tritici	Baihua	China	EU924747	-	-
	R205	Iran	EU014048	-	-
	abc1	Iran	KR230394	-	-
	voucher IRAN 11497 F	Iran	AY956559	-	-
	PA_2	Kentucky, United States	GU598104	-	-
	NYH_4	United States	GU598094	-	-
	JCP58	Mexico, United States	KT982697	-	-
	JCP58	Mexico, United States	KT982690	-	-
	voucher	Sydney	-	EF570842	-
	TU5S	China	-	FJ612005	-
	PST130_8439_8	United States	-	-	HQ698557
P. graminis tritici	R204	Iran	EU014047	-	-
	R203	Iran	EU014046	-	-
	89OR436B	United States	DQ417380	-	-
	68MO192-1A	United States	DQ417379	-	-

	70MEX3A	United States	DQ417377	-	-
	80MN518-3	United States	DQ417376	-	-
	59OH5B	United States	DQ417375	-	-
	57KS17-2C	United States	DQ417374	-	-
	61PA80A	United States	DQ417373	-	-
	2010-001	United States	KJ643593	-	-
	343-1,2,3,5,6	Australia	-	DQ983221	-
P. triticina	JCP21	Mexico, United States	KT982689	-	-
	98EGY151C	United States	DQ417419	-	-
	HSZ0748	United States	DQ417416	-	-
	HSZ0747	United States	DQ417415	-	-
	EGY98A	California, United States	AY187087	-	-
	00LA87	United States	AF511083	-	-
	JCP21	Mexico, United States	KT982689	-	-
	R206	Belgium	EU014050	-	-
	PK-189	Pakistan	KJ934918	-	-
	PK-178	Pakistan	KJ934916	-	-
	voucher B 70 0012410	Canada	-	HM147312	-
	voucher BR 59352-85	Canada	-	HM147311	-
	DAOM 240975	Canada	-	HQ317596	-

	DAOM 240974	Canada	-	HQ317595	-
	voucher	Australia	-	EF570812	-
	BP88134	Hungary	-	HQ317599	-
Uromyces euphorbiae-corniculati	F425	Switzerland	AF180158	-	-
Uromyces pisi	E374	Switzerland	AF180192	-	-
Uromyces viciae-fabae	voucher UME LE307/03	Australia	-	EF570865	-
Uromyces striatus	voucher DAR75753	Australia	-	EF570864	-
