# Supplementary material for: Molecular characterization of Indian pathotypes of Puccinia striiformis f. sp. tritici and multigene phylogenetic analysis to establish inter- and intraspecific relationships
Source: Genet Mol Biol. 2018 Sep 21;41(4):834–42. doi: 10.1590/1678-4685-GMB-2017-0171 (PMC6415613; doi:10.1590/1678-4685-GMB-2017-0171)
Supplement: Supplementary file 3 [file 1415-4757-GMB-1678-4685-GMB-2017-0171-s001.pdf]

Supplementary Material to "Molecular characterization of Indian pathotypes of *Puccinia striiformis* f. sp. *tritici* and multigene phylogenetic analysis to establish inter- and intraspecific relationships"

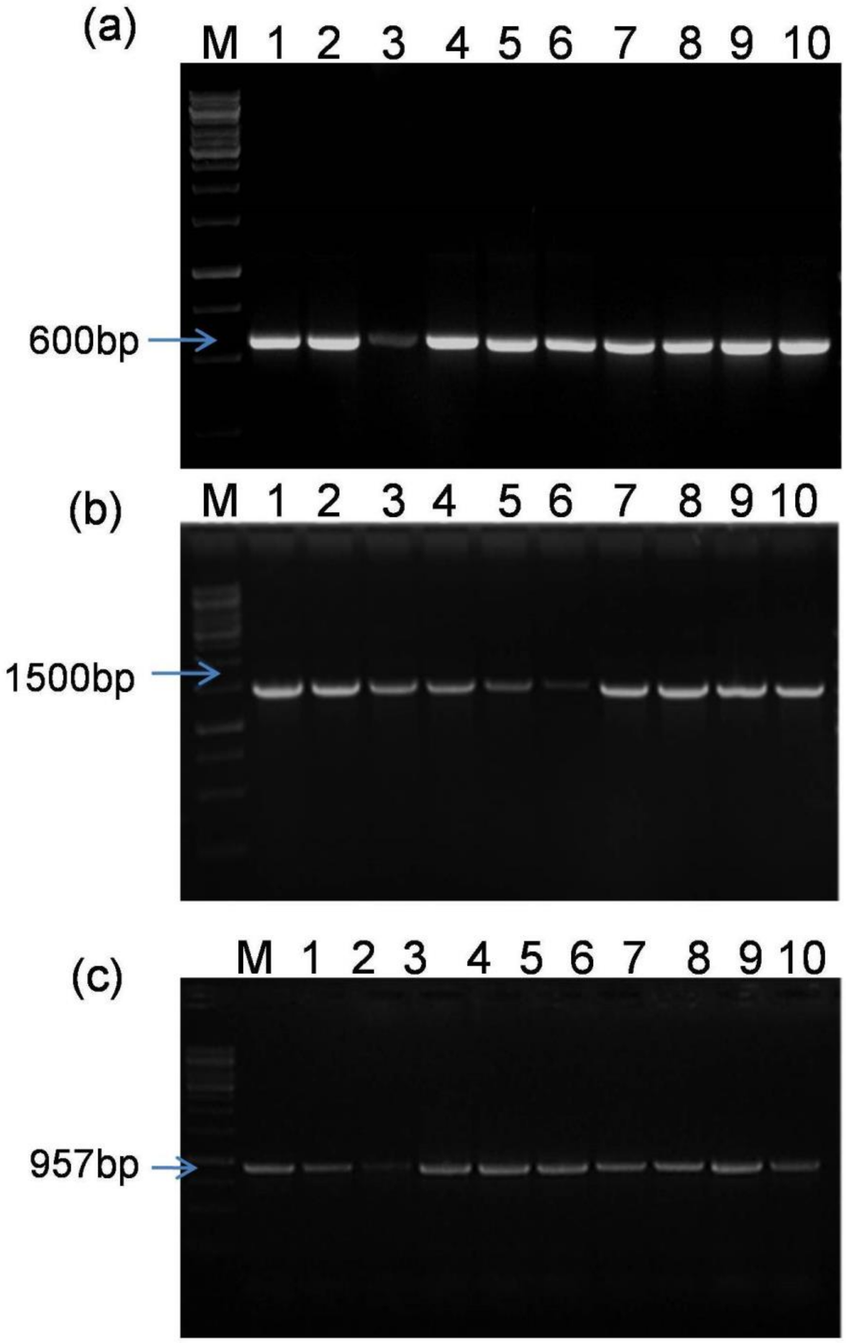

Figure S1 - Agarose gel showing amplified products from polymerase chain reaction using (a) internal transcribed spacer region (ITS), (b) *ketopantoate reductase,* and (c) *β-tubulin* gene specific primers with genomic DNA of different races of *P. striiformis* f. sp. *tritici*. M:1kb marker; 1-66S64-1; 2-70S0-2; 3-47S102; 4-78S84; 5-46S119; 6-70S4; 7-70S64; 8-67S64; 9-38S102; 10-66S0.
