# Supplementary material for: Molecular characterization of Indian pathotypes of Puccinia striiformis f. sp. tritici and multigene phylogenetic analysis to establish inter- and intraspecific relationships
Source: Genet Mol Biol. 2018 Sep 21;41(4):834–42. doi: 10.1590/1678-4685-GMB-2017-0171 (PMC6415613; doi:10.1590/1678-4685-GMB-2017-0171)
Supplement: Supplementary file 4 [file 1415-4757-GMB-1678-4685-GMB-2017-0171-s002.pdf]

Supplementary Material to "Molecular characterization of Indian pathotypes of *Puccinia striiformis* f. sp. *tritici* and multigene phylogenetic analysis to establish inter- and intraspecific relationships"

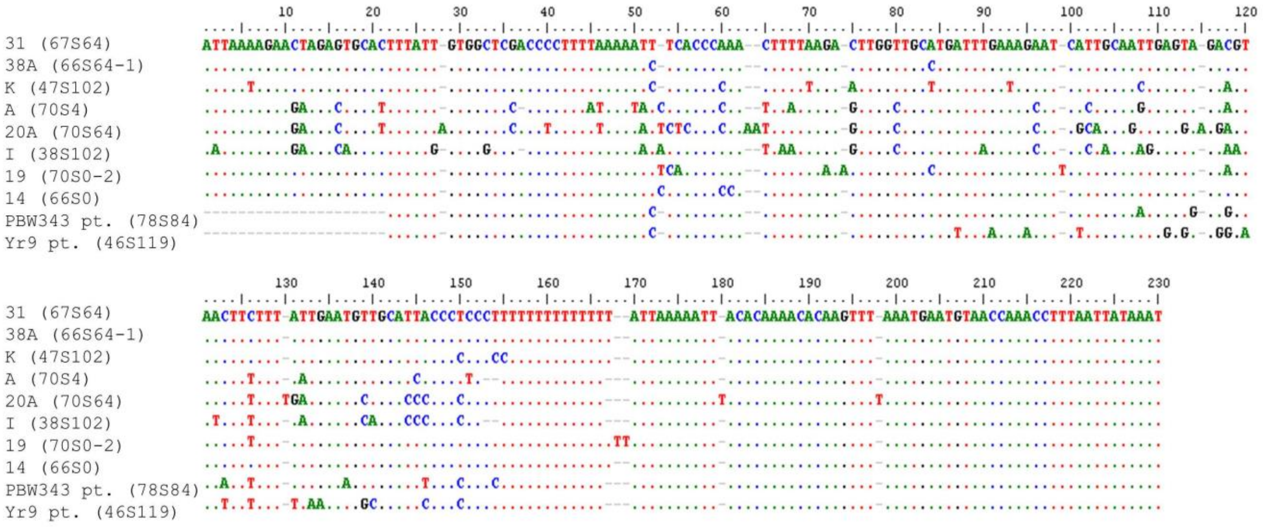

Figure S2 - Multiple sequence alignment of internal transcribed spacer 1 (ITS1) region of 10 Indian pathotypes of *P. striiformis.*
